# Epitope-Based Peptide Vaccine Design against Fructose Bisphosphate Aldolase of *Candida glabrata*: An Immunoinformatics Approach

**DOI:** 10.1155/2021/8280925

**Published:** 2021-05-04

**Authors:** Lina Mohamed Elamin Elhasan, Mohamed B. Hassan, Reham M. Elhassan, Fatima A. Abdelrhman, Essam A. Salih, Asma Ibrahim H, Amna A. Mohamed, Hozaifa S. Osman, Marwa Saad M. Khalil, Athar A. Alsafi, Abeer Babiker Idris, Mohamed A. Hassan

**Affiliations:** ^1^Faculty of Science and Technology, Department of Biotechnology, Omdurman Islamic University, Khartoum, Sudan; ^2^Faculty of Medicine and Health Science, Omdurman Islamic University, Khartoum, Sudan; ^3^Department of Pharmaceutical Chemistry, Faculty of Pharmacy, Sudan International University, Khartoum, Sudan; ^4^Department of Biotechnology, Africa City of Technology, Khartoum, Sudan; ^5^Biology and Technology Department, College of Applied and Industrial Sciences, University of Bahri, Khartoum, Sudan; ^6^Faculty of Pharmacy, National Ribat University, Khartoum, Sudan; ^7^Al-Neelain Medical Research Center, Al-Neelain University, Khartoum, Sudan; ^8^Department of Medical Microbiology, Faculty of Medical Laboratory Sciences, University of Khartoum, Khartoum, Sudan; ^9^Department of Translation Bioinformatics, Detavax Biotech, Kayseri, Turkey

## Abstract

**Background:**

*Candida glabrata* is a human opportunistic pathogen that can cause life-threatening systemic infections. Although there are multiple effective vaccines against fungal infections and some of these vaccines are engaged in different stages of clinical trials, none of them have yet been approved by the FDA.

**Aim:**

Using immunoinformatics approach to predict the most conserved and immunogenic B- and T-cell epitopes from the fructose bisphosphate aldolase (Fba1) protein of *C. glabrata*. *Material and Method*. 13 *C. glabrata* fructose bisphosphate aldolase protein sequences (361 amino acids) were retrieved from NCBI and presented in several tools on the IEDB server for prediction of the most promising epitopes. Homology modeling and molecular docking were performed.

**Result:**

The promising B-cell epitopes were AYFKEH, VDKESLYTK, and HVDKESLYTK, while the promising peptides which have high affinity to MHC I binding were AVHEALAPI, KYFKRMAAM, QTSNGGAAY, RMAAMNQWL, and YFKEHGEPL. Two peptides, LFSSHMLDL and YIRSIAPAY, were noted to have the highest affinity to MHC class II that interact with 9 alleles. The molecular docking revealed that the epitopes QTSNGGAAY and LFSSHMLDL have the lowest binding energy to MHC molecules.

**Conclusion:**

The epitope-based vaccines predicted by using immunoinformatics tools have remarkable advantages over the conventional vaccines in that they are more specific, less time consuming, safe, less allergic, and more antigenic. Further in vivo and in vitro experiments are needed to prove the effectiveness of the best candidate's epitopes (QTSNGGAAY and LFSSHMLDL). To the best of our knowledge, this is the first study that has predicted B- and T-cell epitopes from the Fba1 protein by using in silico tools in order to design an effective epitope-based vaccine against *C. glabrata*.

## 1. Introduction

Candidiasis is a fungal infection that has a high burden of morbidity and mortality in hospitalized and immunocompromised patients. It occurs in more than a quarter of a million patients every year with incidence rates for candidemia of 2–14 per 100000 [1–4]. In general, Candida species infection ranges from superficial mucosal candidiasis such as vulvovaginal candidiasis and oropharyngeal candidiasis to serious systemic infection such as candidemia or fungemia [5–8]. Pathogenicity is facilitated by a number of virulence factors, most importantly its ability to adhere to host surfaces including medical devices, biofilm formation, and secretion of hydrolytic enzymes. Also, Candida cells elaborate polysaccharides, proteases, phospholipases, and hemolysins that cause host cell damage which leads to the increase in the incidence and antifungal resistance of NCAC species, specifically *C. glabrata*, and the unfortunate high morbidity and mortality associated with these species [8, 9].

*Candida glabrata* (*C. glabrata*) is a human opportunistic pathogen that can cause life-threatening systemic infections. *C. glabrata* is not polymorphic, grows as blastoconidia (yeast), and lacks pseudohyphal formation, so it is classified in the genus Torulopsis. *C. glabrata* cells (1–4 *μ*m in size) forms glistening, smooth, and cream-colored colonies [10, 11]. During the infection, *C. glabrata* pathogens invade the macrophages, which are considered part of the innate immune system which is the first line of defense against invading pathogens. *C. glabrata* is able to modify the macrophage's phagosomal compartment, avoiding full maturation and acidification, and thus prevents the forming of the phagolysosomal environment [12]. *C. glabrata* is able to invade the bloodstream and different organs in a mouse model that have intragastrointestinal infections [13].

*C. glabrata* has a haploid genome—published in 2004 by Dujon et al. [14]—that allows adaptation to a wide range of environments [9, 15, 16]. Also, its genome contains more tandem repeats of genes than the other Nakaseomyces [17] and covers 67 genes encoding putative adhesin (cell wall proteins), including the Epa family with 17 members [16, 18], such as epithelial adhesin 1 (Epa1p) [19] and fructose bisphosphate aldolase protein (Fba1) which play an essential role in the pathogenicity of Candida species mainly in the adhesion of the pathogen to the host [20, 21].

Fba1 is a yeast cell wall protein which presents in multiple species of Candida, e.g., *C. glabrata*, *C. parapsilosis*, *C. tropicalis*, and *C. albicans* fungal pathogens [22–26]. Fba1 is an important enzyme in the glycolytic pathway [27–30] and is also a multifunctional protein [31] that can facilitate the attachment (adhesion) to human cells or abiotic surfaces [32–34], protects Candida cells from the host's immune system [33], and promotes the detoxification of the ROS generated during the respiratory burst [21, 33, 34]. However, proteomics analysis revealed that Fba1 is the most abundant and stable enzyme in Candida. Moreover, it is considered one of the main immunodominant proteins [35, 36] in Candida cells and has been tested in the murine model as a protected protein against Candida [37], especially *C. albicans*, and also introduced immunity to *C. glabrata* [34]; therefore, Fba1 is a potential antifungal target in yeast [38]. Multiple vaccines used Fba1 as an immunogenic protein against different pathogens such as the lethal and challenging *S. pneumoniae*, *Salmonella* spp., and *M. bovis* [39, 40].

The incidence of fungal infection has been increasing in the last few years, due to several factors such as misuse of broad-spectrum antibiotics, cytotoxic chemotherapy, immunocompromised patients, and transplantations [15, 41]. Invasive fungal infections are a major cause of global morbidity and mortality, accounting for about 1.4 million deaths per year [42]. Systemic fungal infections cost the healthcare industry approximately $2.6 billion per year in the USA alone [43]. However, Candida species pose a base problem in hospitals, according to Healthcare-Associated Infections (HAI) [19, 44–46]. Although there are multiple effective vaccines against fungal infections and some of these vaccines are engaged in different stages of clinical trials, none of them have yet been approved by the FDA [47]. Therefore, there is an urgent and crucial need to design vaccines against the Candida species that might improve the quality of life for immunosuppressed patients [48].

The aim of this study is to predict the most conserved and immunogenic B- and T-cell epitopes from the Fba1 protein of *C. glabrata* by using in silico tools with the immunoinformatics approach presented in the IEDB server [49, 50]. This approach has multiple benefits in comparison to other approaches by being affordable, safe, time-saving, and clinically applicable using different computational software techniques [51–53]. To the best of our knowledge, this is the first study that has predicted the best candidates of multiple epitopes for Fba1 protein against *C. glabrata*.

## 2. Materials and Methods

**Figure 1:**
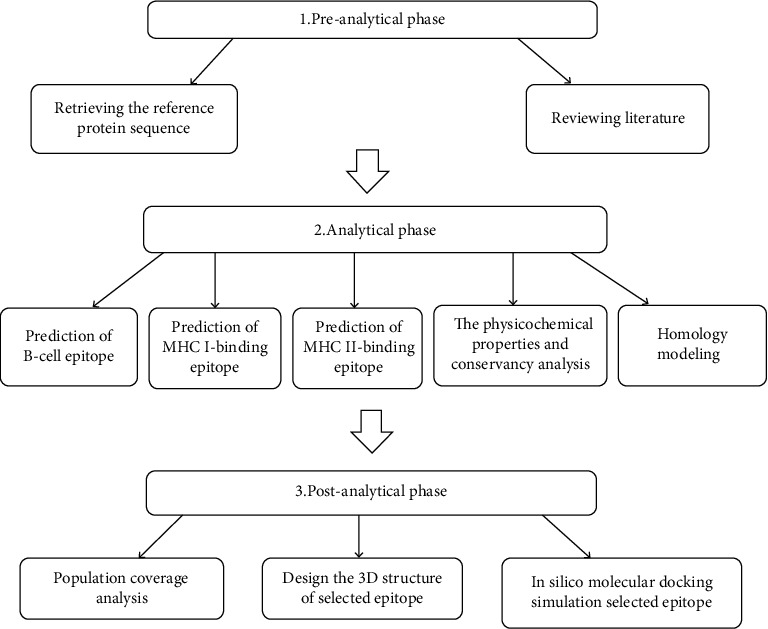
Schematic representation of the methodology phases.

In this study, we have used a variety of bioinformatics databases and tools for the prediction of the most promising peptides, through three phases shown in Figure 1.

### 2.1. Retrieval of Fructose Bisphosphate Aldolase Protein Sequences

13 *Candida glabrata* fructose bisphosphate aldolase protein sequences (361 amino acids) were retrieved from the NCBI (https://www.ncbi.nlm.nih.gov/protein) database on 21 January 2019. The accession numbers of fructose bisphosphate aldolase protein sequences were CAG61849.1, XP_448879.1, KTB01194.1, KTB08502.1, KTB09791.1, KTB12564.1, KTB19354.1, KTB25695.1, KTB27082.1, OXB40821.1, OXB46121.1, SLM13767.1, and SCV14850.1 [20].

### 2.2. Determination of Conserved Regions

Multiple sequence alignment (MSA) was used to determine the conserved regions; the retrieved sequences were aligned by MSA using Clustal W as applied in the BioEdit [54].

### 2.3. Prediction of B-Cell Epitope

The reference sequence of fructose bisphosphate aldolase protein was submitted to the following B-cell tests [49, 50].

#### 2.3.1. Prediction of Linear B-Cell Epitopes

A collection of methods to predict linear B-cell epitopes based on protein sequence characteristics of the antigen using amino acid scales and HMMs was used.

The Bepipred tool from IEDB (http://tools.iedb.org/bcell/result/) was used to predict the linear B-cell epitopes from the conserved region with a default threshold value of 0.350 [55–57].

#### 2.3.2. Prediction of Surface Accessibility

Emini surface accessibility prediction tool of the Immune Epitope Database (IEDB) (http://tools.iedb.org/bcell/result/) was used to tupredict the surface epitopes from the conserved region with the default threshold value 1.0 [58].

#### 2.3.3. Prediction of Epitope Antigenicity

The Kolaskar and Tongaonkar antigenicity method was used to detect the antigenic sites with a default threshold value of 1.025 (http://tools.iedb.org/bcell/result/) [59].

#### 2.3.4. Prediction of Discontinuous B-Cell Epitopes

This method predicts epitopes based upon solvent-accessibility and flexibility. The methods are for modeling, docking of antibody, and protein 3D structures (http://tools.iedb.org/bcell/result/).

The modeled 3D structure was submitted to the ElliPro (http://tools.iedb.org/ellipro/) prediction tool to filter out the antigenic residues. The minimum score and maximum distance (Angstrom) were calibrated in the default mode with a score of 0.5 and 6, respectively [60].

### 2.4. Prediction of MHC Class I Binding Epitopes

The peptides' binding affinity to MHC I molecules was defined by the IEDB MHC I prediction tool at http://tools.iedb.org/mhc1. The binding affinity of fructose bisphosphate aldolase peptides to MHC1 molecules was obtained using the artificial neural network (ANN) method. All conserved epitopes that bind to MHC1 alleles at score ≤ 500 half-maximal inhibitory concentrations (IC50) with peptides that have a length of 9 amino acids were selected for further analysis [49, 61–66].

### 2.5. MHC Class II Binding Predictions

Prediction of peptide binding affinity to MHC II molecules was defined by the IEDB MHC II prediction tool at http://tools.iedb.org/mhcii/result/. MHC II molecules have the ability to bind peptides with different lengths which make the prediction accuracy debatable. For MHC II binding predication, human allele reference sets were used. The prediction method was selected as NN-align to asses both the binding affinity and MHC II binding core epitopes with a length of 9 amino acid peptides at score IC50 of 100 [49, 67].

### 2.6. Population Coverage Calculation

The candidate epitopes of MHC I and MHC II and combined binding of MHC I and MHC II alleles from *Candida glabrata* fructose bisphosphate aldolase protein were employed for population coverage, and the world population was set as a target population for the selected MHC I and MHC II combined binding alleles using the IEDB population coverage calculation tool at http://tools.iedb.org/population/ [49, 68].

### 2.7. Homology Modeling

The reference sequence of *Candida glabrata* fructose bisphosphate aldolase protein was applied to Raptor X for modeling at http://raptorx.uchicago.edu/. Then, the 3D structural model of the protein was visualized by using the Chimera tool powered by UCSF [69–73].

### 2.8. Physicochemical Parameters

The function of vaccines is to enhance the immunogenic response once introduced to the immune system. Thus, it is essential to recognize the physicochemical parameters of the protein using the protein protogram and BioEdit [54] (available at https://web.expasy.org/protparam/ and https://web.expasy.org/protscale/) [74].

### 2.9. Molecular Docking Analysis

Molecular docking was performed using Moe 2007. The 3D structures of the promiscuous epitopes were predicted by PEP-FOLD. The crystal structures of HLA-A∗02:06 (*PDB ID 3OXR*) and HLA-DRB1∗01:01 (*PDB ID 5JLZ*) were chosen as a model for molecular docking and were downloaded in a PDB format from the RCSB PDB resource. However, the selected crystal structures were in a complex form with ligands. Thus, to simplify the complex structure of all water molecules, hetero groups and ligands were removed by Discovery Studio Visualizer 2.5. Partial charge and energy minimization were applied for ligands and targets. In terms of the identification of the binding groove, the potential binding sites in the crystal structure were recognized using the Alpha Site Finder. Finally, ten independent docking runs were carried out for each peptide. The results were retrieved as binding energies. Best poses for each epitope that displayed the lowest binding energies were visualized using UCSF Chimera 1.13.1 software [72, 75–78].

## 3. Result

### 3.1. B-Cell Epitope Prediction

**Figure 2:**
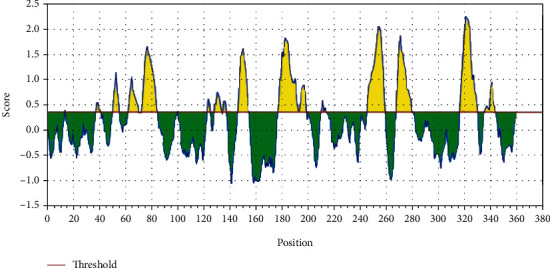
Bepipred linear epitope prediction: the red line is the threshold; above (the yellow part) is proposed to be part of the B-cell epitope.

The reference sequence of fructose bisphosphate aldolase from *C. glabrata* was analyzed using a Bepipred linear epitope prediction test; the average binder's score of the protein to B-cell was 0.199 and minimum was -0.009 and 2.424 for a maximum score; all values equal or greater than the default threshold 0.350 which were potentially linear epitopes are shown in Figure 2.

#### 3.1.1. Prediction of Surface Accessibility

**Figure 3:**
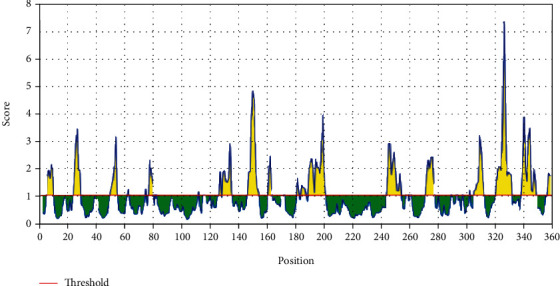
Emini's surface accessibility prediction test: the red line is the threshold; above (the yellow part) is proposed to be part of the B-cell epitope.

In Emini's surface accessibility prediction test, for a potent B-cell epitope, the average surface accessibility area of the Fba1 protein was scored as 1.000, with a maximum of 7.725 and a minimum of 0.113; all values equal or greater than the default threshold 1.000 were potentially in the surface shown in Figure 3.

#### 3.1.2. Prediction of Epitope Antigenicity

**Figure 4:**
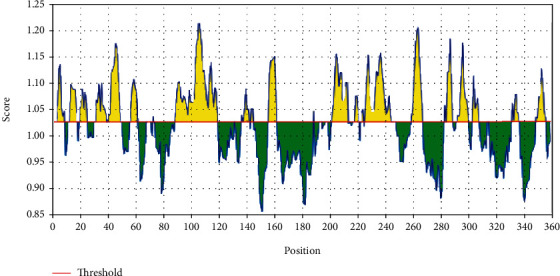
Kolaskar and Tongaonkar antigenicity prediction test: the red line is the threshold; above (the yellow part) is proposed to be part of the B-cell epitope.

For the Kolaskar and Tongaonkar antigenicity prediction test, the average of antigenicity was 1.025, with a maximum of 1.223 and a minimum of 0.853; all values equal to or greater than the default threshold 1.025 are potential antigenic determinants (see Figure 4). The results of all proposed conserved predicted B-cell epitopes are shown in Table 1. The list of the most promising B-cell epitopes with their surface scores and antigenicity is shown in Table 2.

#### 3.1.3. Discontinuous B-Cell Epitope Prediction

The modeled 3D structure of the Fba1 protein was submitted to the ElliPro prediction tool to filter out the antigenic residues. The minimum score and maximum distance (Angstrom) were calibrated in the default mode with a score of 0.5 and 6, respectively (see Table 3 for more illustrations).

### 3.2. T-Cell Peptide Prediction

#### 3.2.1. Prediction of MHC I Binding Profile for T Cytotoxic Cell Conserved Epitopes

114 epitopes were anticipated to interact with different MHC I alleles. The core epitopes KYFKRMAAM and QTSNGGAAY were noticed to be the dominant binders with 7 alleles for each (HLA-A∗24:02, HLA-A∗30:01, HLA-A∗31:01, HLA-B∗14:02, HLA-C∗07:02, HLA-C∗12:03, and HLA-C∗14:02) (HLA-A∗01:01, HLA-A∗26:01, HLA-A∗29:02, HLA-A∗30:02, HLA-B∗15:01, HLA-B∗15:02, and HLA-B∗35:01) followed by AVHEALAPI, RMAAMNQWL, and YFKEHGEPL which bind with five alleles; these findings are shown in Table 4.

#### 3.2.2. Prediction of MHC II Binding Profile for T Helper Cell Conserved Epitopes

102 conserved predicted epitopes were found to interact with MHC II alleles. The core epitope LFSSHMLDL is thought to be the top binder as it interacts with 9 alleles (HLA-DRB1∗07:01, HLA-DPA1∗01, HLA-DPB1∗04:01, HLA-DPA1∗01:03, HLA-DPB1∗02:01, HLA-DPA1∗02:01, HLA-DPB1∗01:01, HLA-DPA1∗03:01, and HLA-DPB1∗04:02), followed by IRGSIAAAH which binds to five alleles and VVAALEAAR which also binds with five alleles but with low frequency. Followed by YQAGNVVLS and IAPAYGIPV, these findings are shown in Table 5.

### 3.3. Population Coverage

The most interesting findings in this test is the population coverage analysis result for the most common binders to MHC I and MHC II alleles each and combined among the world, exhibiting an exceptional coverage with percentages 92.54%, 99.58%, and 98.5%, respectively.

#### 3.3.1. Population Coverage for Isolated MHC I

**Figure 5:**
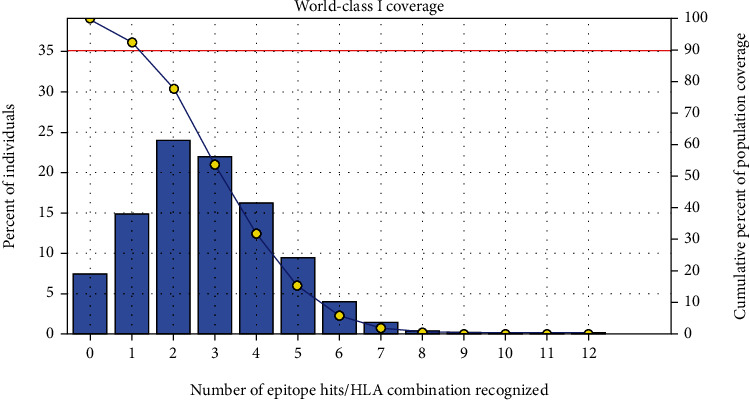
Global coverage for the top five MHC I peptides (AVHEALAPI, KYFKRMAAM, QTSNGGAAY, RMAAMNQWL, and YFKEHGEPL). Note: in the graph, the line (-o-) represents the cumulative percentage of population coverage of the epitopes; the bars represent the population coverage for each epitope.

Five epitopes are given to interact with the most frequent MHC class I alleles: *AVHEALAPI*, *KYFKRMAAM*, *QTSNGGAAY*, *RMAAMNQWL*, and *YFKEHGEPL*, representing a considerable coverage against the whole world population. The maximum population coverage percentage over these epitopes is 92.54% (see Figure 5).

#### 3.3.2. Population Coverage for Isolated MHC II

**Figure 6:**
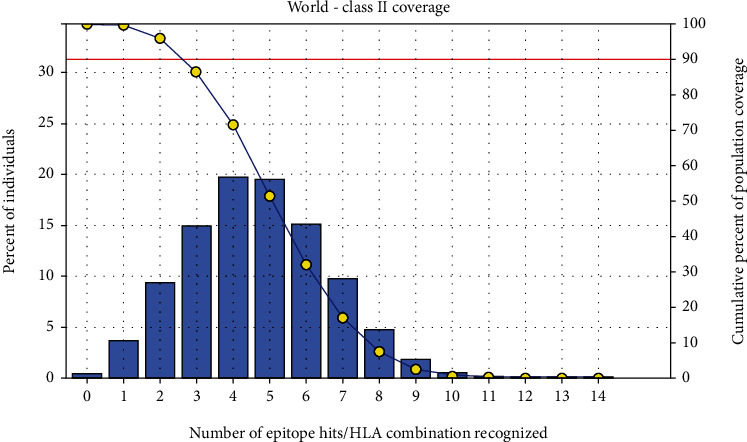
Global proportion for the top five MHC II IRGSIAAAH, LFSSHMLDL, and VVAALEAAR. Notes: in the graph, the line (-o-) represents the cumulative percentage of population coverage of the epitopes; the bars represent the population coverage for each epitope.

Three epitopes were assumed to interact with the most frequent MHC class II alleles (IRGSIAAAH, LFSSHMLDL, and VVAALEAAR) with a percentage of 99.58%. The *LFSSHMLDL* epitope shows an exceptional result for the population coverage test for MHC II binding affinity of 96.60% globally (see Figure 6).

#### 3.3.3. Population Coverage for MHC I and MHC II Alleles Combined

**Figure 7:**
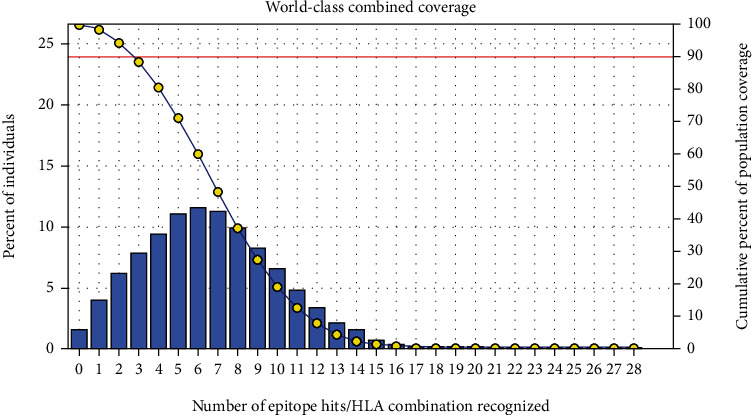
Global population proportion for the top five MHC I and II epitopes in combined mode (IAPAYGIPV, AAFGNVHGV, VVAALEAAR, YIRSTIAPAY, and YQAGMVVLS). Notes: in the graphs, the line (-o-) represents the cumulative percentage of population coverage of the epitopes; the bars represent the population coverage for each epitope.

Regarding the combined MHC I and MHC II alleles, five epitopes were supposed to interact with the most predominant MHC class I and MHC class II alleles (IAPAYGIPV, AAFGNVHGV, VVAALEAAR, YIRSTIAPAY, and YQAGMVVLS), representing a significant global coverage by the IEDB population coverage tool which revealed coverage with percentage of 98.50% as shown in Figure 7.

### 3.4. Homology Modeling

**Figure 8:**
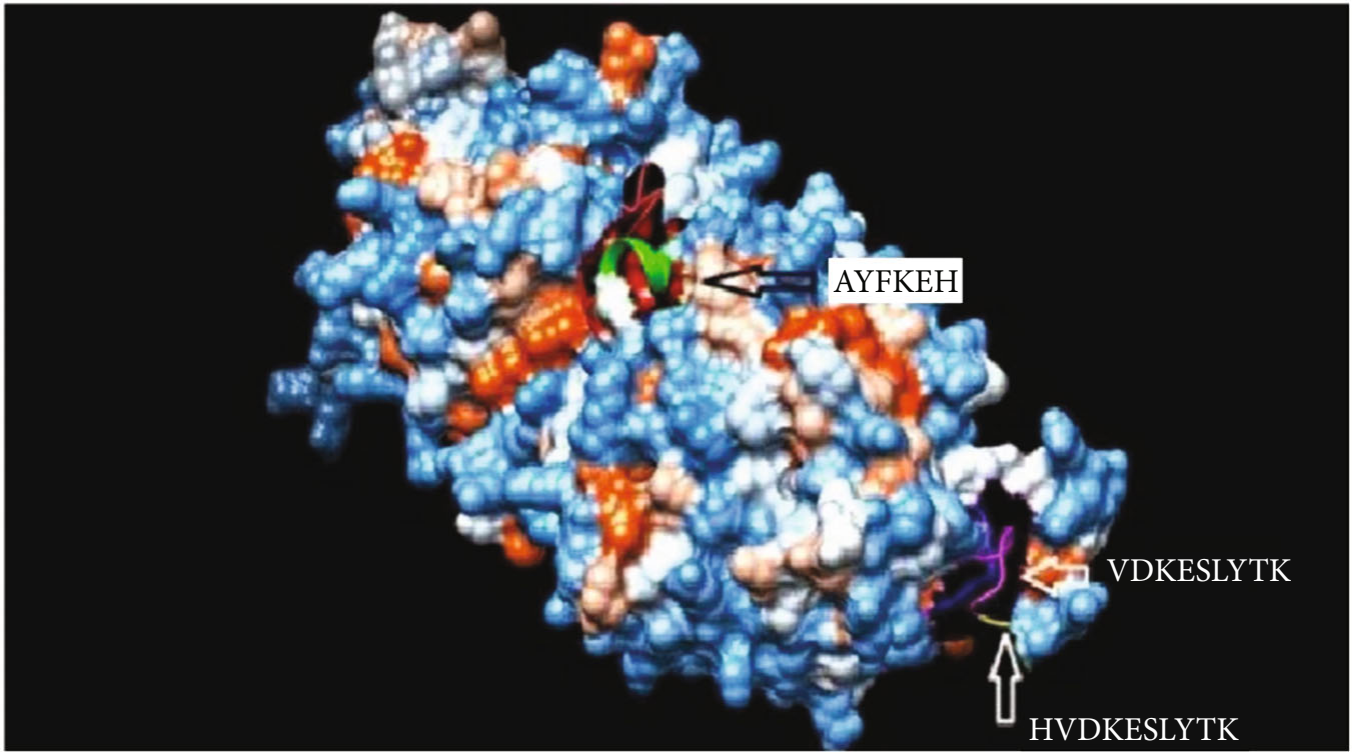
Structural position of the promising B-cell epitope (AYFKEH (in purple color), VDKESLYTK (in yellow color), and HVDKESLYTK (in red color)) in 3-dimensional structure of the fructose bisphosphate aldolase protein from *C. glabrata* using Chimera tool powered by UCSF.

The 3-dimentional structure of the fructose bisphosphate aldolase protein from C*. glabrata* and the most promising peptides binding to MHC class II by using the Chimera tool powered by UCSF are shown in Figure 8.

### 3.5. Physicochemical Parameters

**Figure 9:**
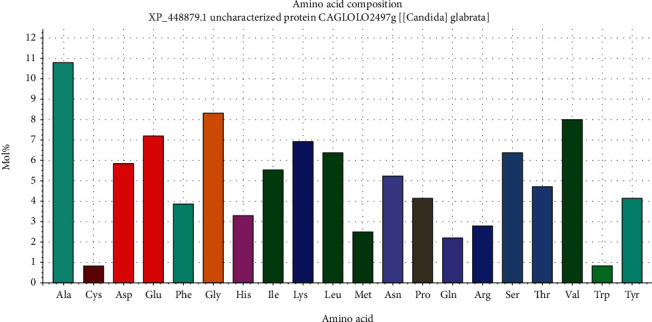
Graph showing amino acid composition of fructose bisphosphate aldolase protein and their molecular weights using BioEdit software 7.0.5.3.

The length of fructose bisphosphate aldolase protein is 361 amino acids, and its molecular weight is 39356.3. Theoretical pI is 5.49 which explain the pH of the protein. Total numbers of negatively and positively charged residues that contain the fructose bisphosphate aldolase protein are (Asp+Glu): 47 and (Arg+Lys): 35, respectively. Also, the number of atoms that compose this protein is 5488 which presented as flowing: carbon 1752, hydrogen 2716, nitrogen 470, oxygen 538, and sulfur 12. N-terminal of the sequence considered is M (Met). The half-life of the fructose bisphosphate aldolase protein estimate is 30 hours (mammalian reticulocytes, in vitro) and more than 20 hours (yeast, in vivo). The aliphatic index and the grand average of hydropathicity (GRAVY) value of vaccine were determined as 80.55 and −0.264, respectively. Instability of the fructose bisphosphate aldolase protein is computed to be 29.93, meaning the protein is stable [74]. The amino acids that compose the protein fructose bisphosphate aldolase with their molecular weights are shown in Table 6 and Figure 9.

### 3.6. Molecular Docking

The best epitopes that displayed the lowest binding energies visualized by using UCSF chimera 1.13.1 software are shown in Table 7 and Figures 10–25.

## 4. Discussion

In the present study, we predicted the most conserved and immunogenic B- and T-cell epitopes from Fba1 protein of *C. Glabrata* by using the immunoinformatics approach in order to develop an effective epitope-based vaccine against this fungal pathogen which has emerged in recent years as a serious health problem especially among immunosuppressed and hospitalized patients [7]. A previous study conducted by de Klerk et al. [79] showed that the Fba1 protein has the ability to provoke immune responses in human against *M. mycetomatis* [79]. Also, several recent publications have used the Fba1 protein as a strong antigenic target for predicting B- and T-cell epitopes in order to design promising vaccines against fungal and bacterial pathogens such as *M. mycetomatis*, *P. aeruginosa*, *L. monocytogenes*, and *S. mansoni* by using in silico tools [80–83]. Hence, there are more studies to explore the fructose bisphosphate aldolase protein immunogenic role and the possibility to find common conserved epitopes for different organisms.

The principle of using a cocktail of B- and T-cell epitopes in the epitope-based vaccine to trigger humoral as well as cellular mediated immune response is very promising to clear infection instead of humoral or cellular immunity alone, and it was applied before to enhance protection against different kinds of infectious diseases [84, 85]. In this study, the analysis of the Fba1 protein revealed 11 effective epitopes for B-cells (AYFKEH, VDKESLYTK, and HVDKESLYTK) and T-cells (AVHEALAPI, KYFKRMAAM, QTSNGGAAY, RMAAMNQWL, YFKEHGEPL, IRGSIAAAH, LFSSHMLDL, and VVAALEAAR).

However, the molecular docking, which evaluates the binding affinity to MHC molecules [51, 52], showed that the peptides QTSNGGAAY and LFSSHMLDL are the best candidates for designing an effective epitope-based vaccine against *C. glabrata*.

**Figure 10:**
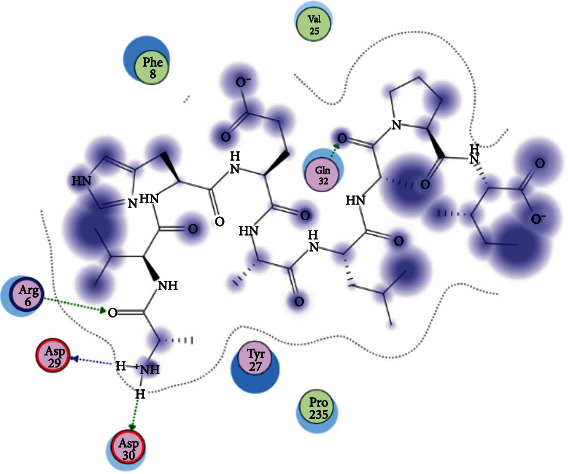
Illustration of the 2D interaction of the best docking poses of AVHEALAPI in the binding sites of HLA-A∗02:06.

**Figure 11:**
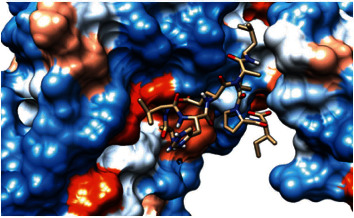
Illustration of the 3D interaction of the best docking poses of AVHEALAPI in the binding sites of HLA-A∗02:06.

**Figure 12:**
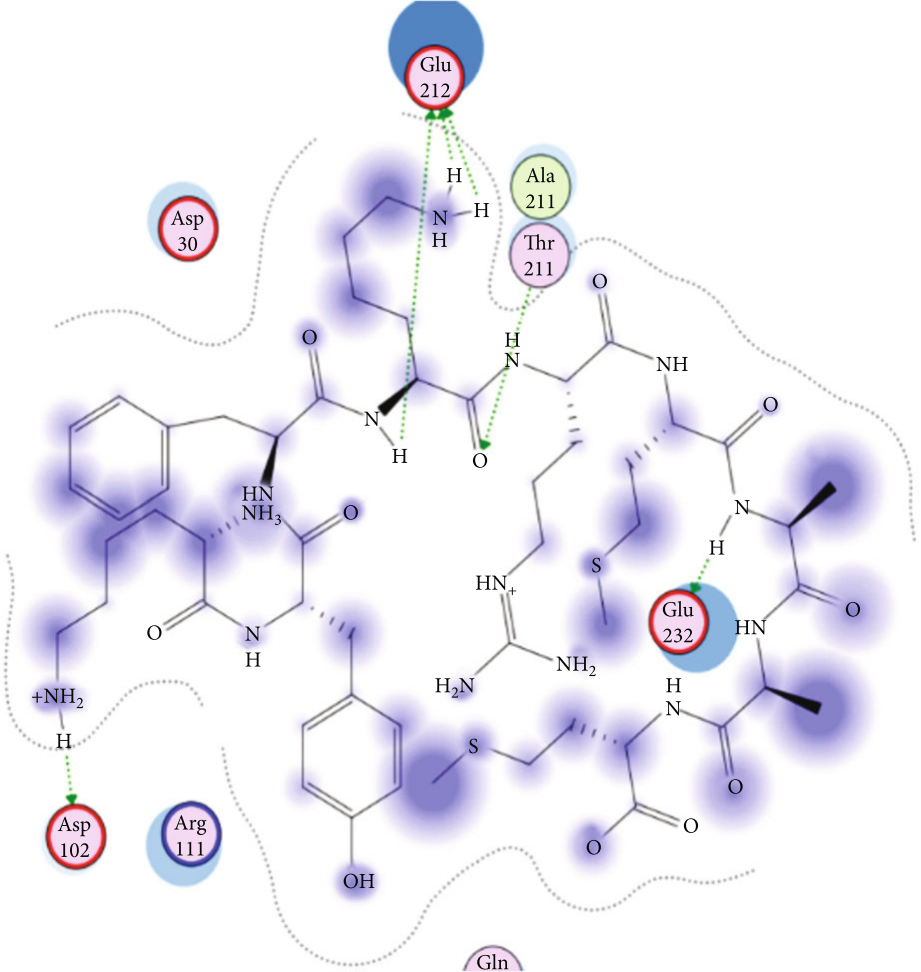
Illustration of the 3D interaction of the best docking poses of KYFKRMAAM in the binding sites of HLA-A∗02:06.

**Figure 13:**
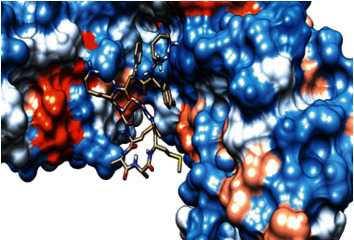
Illustration of the 3D interaction of the best docking poses of KYFKRMAAM in the binding sites of HLA-A∗02:06.

**Figure 14:**
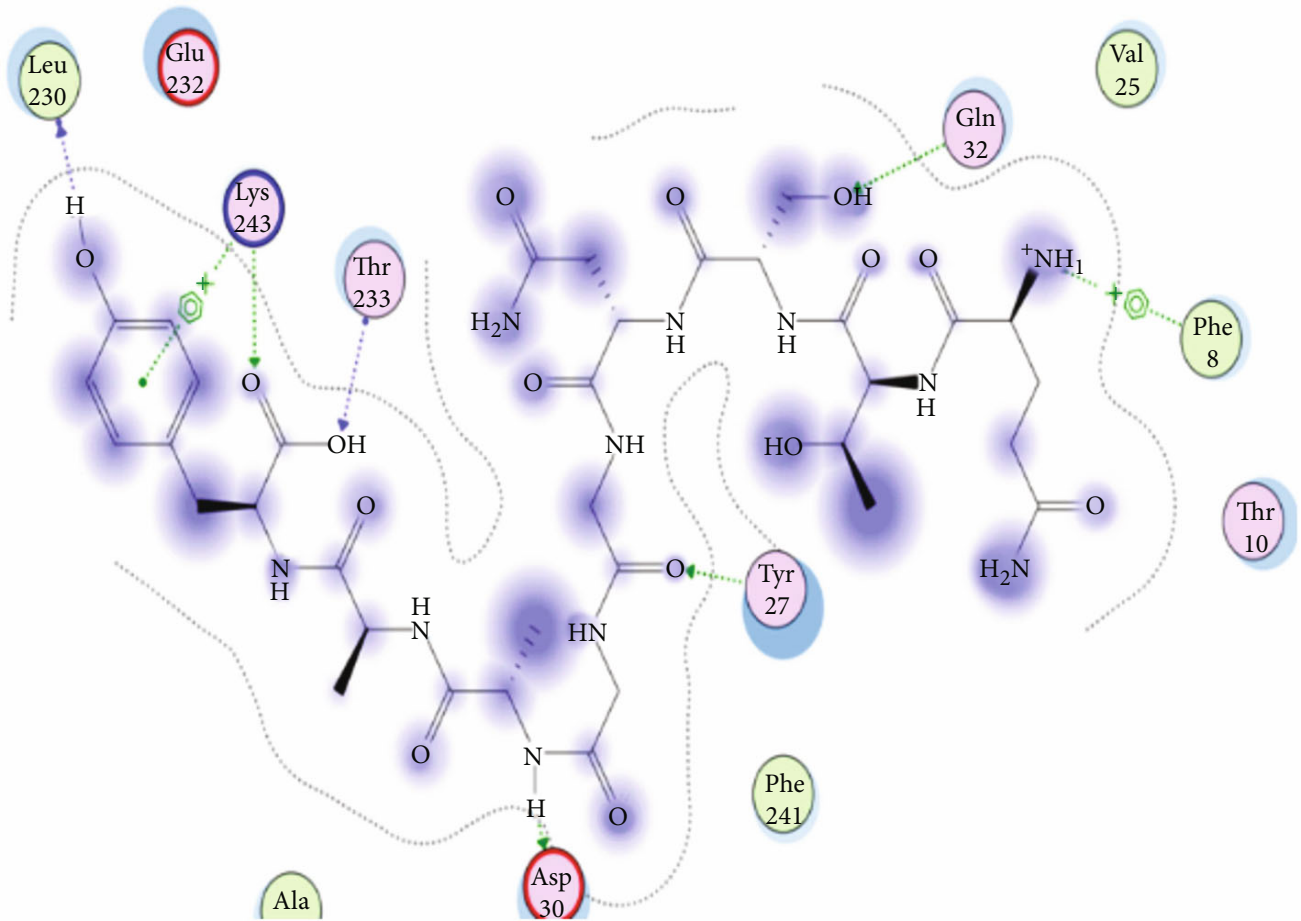
Illustration of the 2D interaction of the best docking poses of QTSNGGAAY in the binding sites of HLA-A∗02:06.

**Figure 15:**
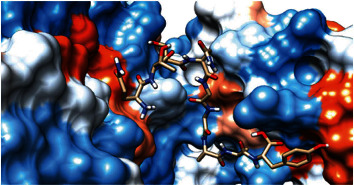
Illustrate the 2D interaction of the best docking poses of QTSNGGAAY in the binding sites of HLA-A∗02:06.

**Figure 16:**
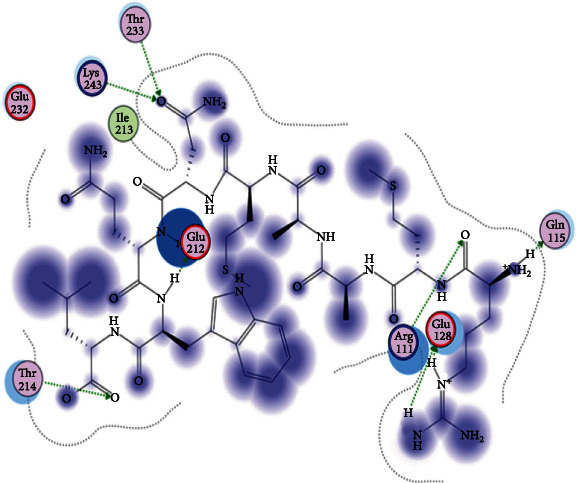
Illustration of the 2D interaction of the best docking poses of RMAAMNQWL in the binding sites of HLA-A∗02:06.

**Figure 17:**
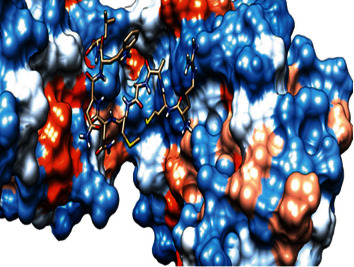
Illustration of the 2D interaction of the best docking poses of RMAAMNQWL in the binding sites of HLA-A∗02:06.

**Figure 18:**
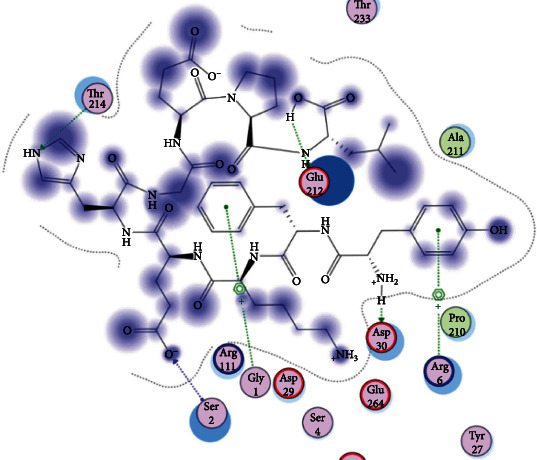
Illustration of the 3D interaction of the best docking poses of YFKEHGEPL in the binding sites of HLA-A∗02:06.

**Figure 19:**
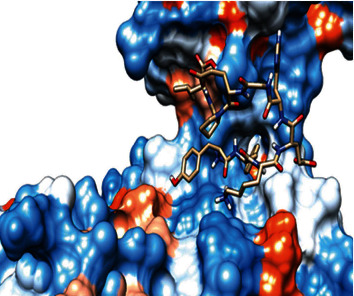
Illustration of the 3D interaction of the best docking poses of YFKEHGEPL in the binding sites of HLA-A∗02:06.

**Figure 20:**
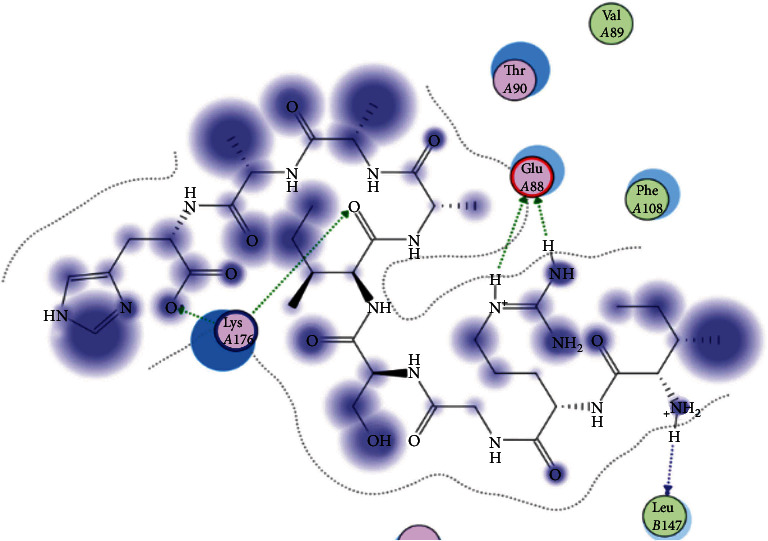
Illustration of the 3D interaction of the best docking poses of IRGSIAAAH in the binding sites of HLA-DRB1∗01:01.

**Figure 21:**
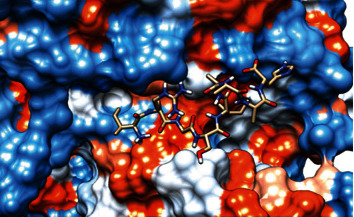
Illustration of the 3D interaction of the best docking poses of IRGSIAAAH in the binding sites of HLA-DRB1∗01:01.

**Figure 22:**
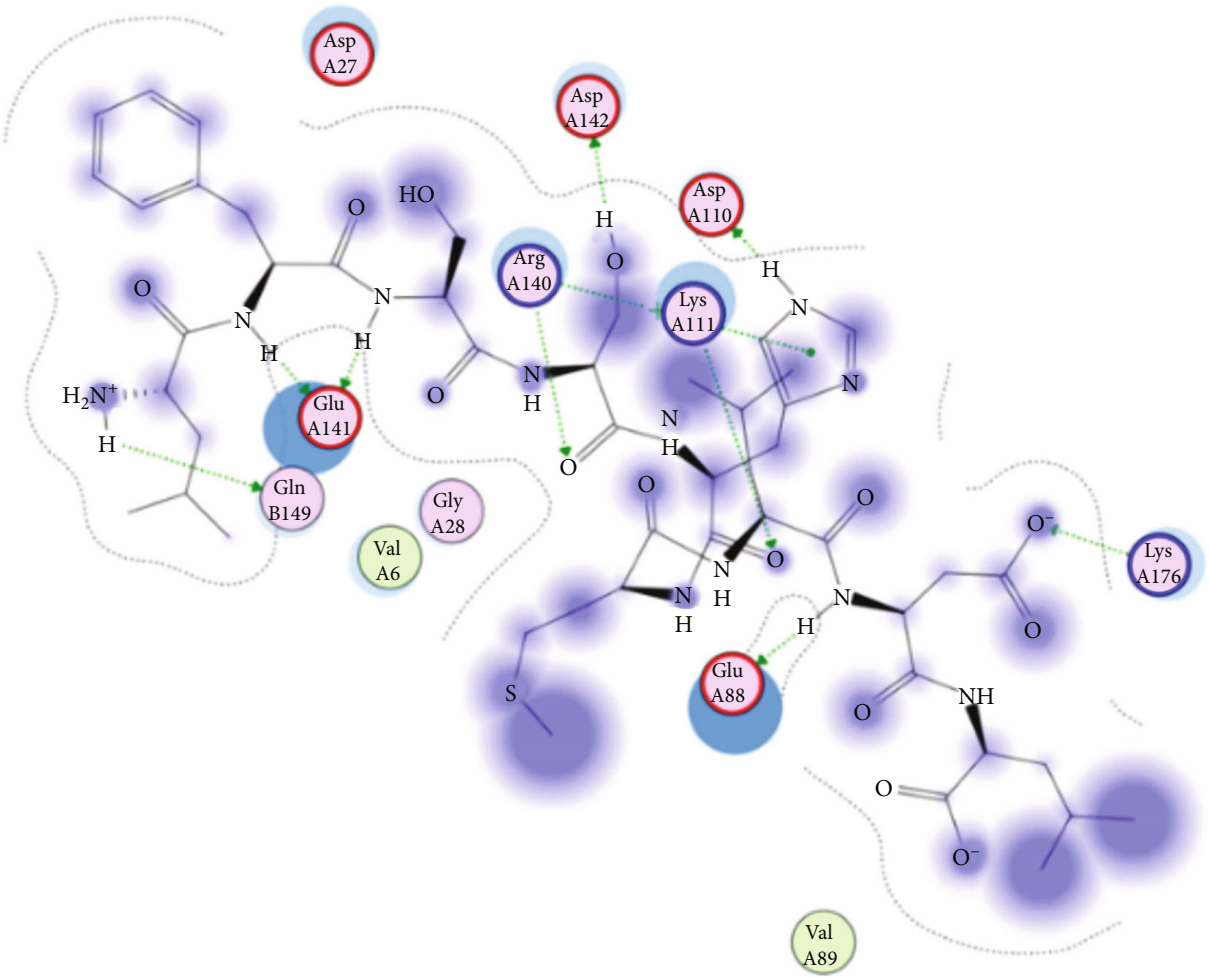
Illustration of the 3D interaction of the best docking poses of LFSSHMLDL in the binding sites of HLA-DRB1∗01:01.

**Figure 23:**
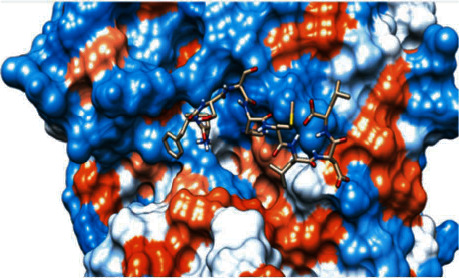
Illustration of the 3D interaction of the best docking poses of LFSSHMLDL in the binding sites of HLA-DRB1∗01:01.

**Figure 24:**
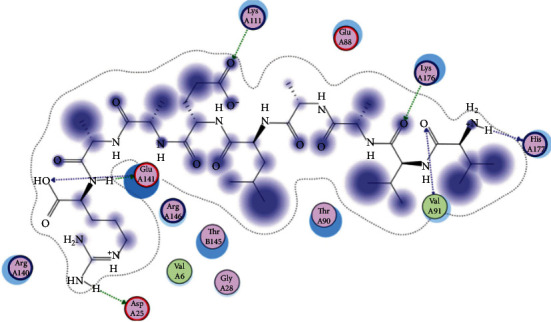
Illustration of the 2D interaction of the best docking poses of VVAALEAAR in the binding sites of HLA-DRB1∗01:01.

**Figure 25:**
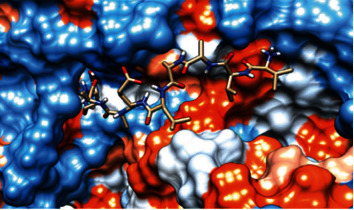
Illustration of the 2D interaction of the best docking poses of VVAALEAAR in the binding sites of HLA-DRB1∗01:01.

After retrieving the various sequences of *C. glabrata* fructose bisphosphate aldolase protein, the protein reference sequence was submitted to the Bepipred linear epitope prediction test, Emini surface accessibility test, and Kolaskar and Tongaonkar antigenicity test in the IEDB, to determine the affinity of B-cell epitopes and their position regarding the surface and their immunogenicity. Three peptides have passed (AYFKEH, VDKESLYTK, and HVDKESLYTK) in all the prediction tests shown in Tables 1 and 2 and Figures 2–4. However, the MHC I binding prediction tool using an artificial neural network (ANN) [61] with half maximal inhibitory concentration (IC50) ≤ 500 revealed 114 conserved peptides interacting with various MHC I alleles. Three peptides were noticed to have the highest affinity in corresponding to their interaction with MHC I alleles. The peptide YIRSIAPAY from 93 to 101 had the affinity with 8 alleles to interact with HLA-A∗26:01, HLA-A∗29:02, HLA-B∗15:01, HLA-A∗30:02, HLA-B∗15:02, HLA-B∗35:01, HLA-C∗14:02, and HLA-C∗12:03, followed in order by KYFKRMAAM from 160 to 168 which interacts with 7 alleles (HLA-A∗24:02, HLA-A∗31:01, HLA-A∗30:01, HLA-B∗14:02, HLA-C∗07:02, HLA-C∗14:02, and HLA-C∗12:03) and QTSNGGAAY from 61 to 69 which interacts with 7 alleles (HLA-A∗01:01, HLA-A∗26:01, HLA-A∗30:02, HLA-A∗29:02, HLA-B∗15:02, HLA-B∗15:01, and HLA-B∗35:01) (see Table 4), while MHC II binding prediction tool using NN-align [67] with half-maximal inhibitory concentration (IC50) ≤ 100 revealed 102 conserved peptides that interact with various MHC II alleles. Two peptides (LFSSHMLDL and YIRSIAPAY) were noted to have the highest affinity in corresponding to their interaction with MHC II alleles; both had the affinity to interact with 9 MHC II alleles (see Table 5). Moreover, the predicted epitopes which have the high affinity to interact with MHC I, MHC II, and combined MHC I with MHC II international alleles were analyzed by population coverage resource in the IEDB [68]. The population coverage of the five most promising epitopes (AVHEALAPI, KYFKRMAAM, QTSNGGAAY, RMAAMNQWL, and YFKEHGEPL) for MHC I alleles was 92.54%, while for the three epitopes (IRGSIAAAH, LFSSHMLDL, and VVAALEAAR) that showed high affinity to MHC II alleles, it was 99.58% throughout the world according to the IEDB database as shown in Figures 5 and 6. It should be noted that the population coverage of the five most promising epitopes that exhibited binding affinity to both MHC I and MHC II alleles (IAPAYGIPV, AAFGNVHGV, VVAALEAAR, YIRSTIAPAY, and YQAGMVVLS) was 98.50% globally (see Figure 7). However, the molecular docking revealed that the epitopes QTSNGGAAY and LFSSHMLDL have high binding energy to MHC molecules HLA-A∗02:06 and HLA-DRB1∗01:01, respectively, which indicate favored affinity and stability in the epitope-molecule complex shown in Table 7 and Figures 10–25. This study was limited by being strictly computational, and more in vitro and in vivo studies to prove the effectiveness of the proposed peptides are highly recommended.

## 5. In Conclusion

The epitope-based vaccines predicted by using immunoinformatics tools have remarkable advantages over the conventional vaccines in that they are more specific, less time consuming, safe, less allergic, and more antigenic. Further in vivo and in vitro experiments are needed to prove the effectiveness of the best candidate's epitopes QTSNGGAAY and LFSSHMLDL. To the best of our knowledge, this is the first study that has predicted B- and T-cell epitopes from the Fba1 protein by using in silico tools in order to design an effective epitope-based vaccine against *C. glabrata*.

## Figures and Tables

**Table 1:** The proposed predicted antigenic B-cell epitopes; 9 antigenic sites were identified from fructose bisphosphate aldolase of *C. glabrata*.

Start	End	Peptide	Length
63	70	*SNGGAAYF*	8
73	84	*KGVSNDGQNASI*	12
129	134	*AYFKEH*	6
147	155	*SEETDDENI*	9
178	199	*ITGGEEDGVNNEHVDKESLYTK*	22
247	260	*KYAAEKTGAPAGSK*	14
269	280	*GSGSTQEEFNTG*	12
318	331	*GNPEGADKPNKKFF*	14
336	345	*WVREGEKTMS*	10

**Table 2:** List of the most promising B-cell epitopes and their surface and antigenicity.

Start	End	Peptide	Length	Surface score (Emini′s surface threshold = 1.000)	Antigenicity score (Kolaskar′s test = 1.025)
129	134	*AYFKEH*	6	1.502	1.034
191	199	*VDKESLYTK*	9	2.48	1.032
190	199	*HVDKESLYTK*	10	2.648	1.04

**Table 3:** List of the promising discontinuous B-cell epitopes.

No.	Residues	Number of residues	Score
1	T300, G301, I302, R303, D304, Y305, V306, L307, N308, K309, K310, D311, Y312, I313, M314, S315, M316, V317, G318, N319, P320, E321, G322, A323, D324, K325, P326, N327, K328, K329, F330, F331, E339, K342	34	0.867
2	D332, P333, R334, V335, W336	5	0.749
3	V3, Q4, E5, V6, L7, K8, Y25, E28, H29, K30, F31, K55, S56, A156, T157, V159, K160, K163, G177, I178, T179, G180, G181, E182, E183, D184, G185, V186, N187, N188, E189, H190, V191, D192, K193, E194, S195, L196, Y197, T198, K199, P200, E201, F204, A205, E208, A209, A211, P212, I213, S214, P215, A222, F223, G224, Q231, A232, G233, N234, V235, V236, L237, S238, P239, E240, A243, D244, K247, Y248, A249, A250, E251, K252, T253, G254, A255, P256, A257, G258, S259, K260, P261, S272, T273, Q274, E275, N278, T279, N282, N283, T357, K358, N359, T360, L361	95	0.669
4	V15, G16, A71, G72, K73, G74, V75, S76, N77, D78, G79, Q80, N81, A82, I84, R85, C112, A113, K114, L117, P118, D121, G122, L124, E125, A126, E128, A129, Y130, F131, K132, E133, H134, G135, E136, P137, L138, R164, A166, A167, M168, N169, Q170	43	0.668
5	L146, S147, E148,E149, T150, D151, D152, E153	8	0.582
6	R9, K10, T11, G12, I14, R52, D53, A98, P99, A100, Y101, G102, I103	13	0.514

**Table 4:** Promising T-cell epitopes (class MHC I alleles) with their position and IC50 value.

Core epitope	Start	End	Allele	IC50
KYFKRMAAM	160	168	HLA-A∗24:02	451.84
	160	168	HLA-A∗30:01	232.12
	160	168	HLA-A∗31:01	131.22
	160	168	HLA-B∗14:02	427.02
	160	168	HLA-C∗07:02	149.13
	160	168	HLA-C∗12:03	240.46
	160	168	HLA-C∗14:02	6.27
AVHEALAPI	205	213	HLA-A∗02:01	154.37
	205	213	HLA-A∗02:06	9.78
	205	213	HLA-A∗30:01	20.96
	205	213	HLA-A∗32:01	122.32
	205	213	HLA-A∗68:02	55.22
RMAAMNQWL	164	172	HLA-A∗02:01	52.44
	164	172	HLA-A∗02:06	237.09
	164	172	HLA-A∗32:01	79.39
	164	172	HLA-B∗15:01	258
	164	172	HLA-C∗14:02	482
QTSNGGAAY	61	69	HLA-A∗01:01	54.18
	61	69	HLA-A∗26:01	89.37
	61	69	HLA-A∗29:02	56.68
	61	69	HLA-A∗30:02	47.89
	61	69	HLA-B∗15:01	111.57
	61	69	HLA-B∗15:02	82.52
	61	69	HLA-B∗35:01	99.45
YFKEHGEPL	130	138	HLA-B∗08:01	295.97
	130	138	HLA-C∗03:03	42.03
	130	138	HLA-C∗07:02	319.29
	130	138	HLA-C∗12:03	26.8
	130	138	HLA-C∗14:02	18.47

**Table 5:** Promising T-cell epitope (class MHC II alleles) with their position and peptide sequence and IC50 value and rank.

Core sequence	Allele	Start	End	Peptide sequence	IC50	Rank
*LFSSHMLDL*	HLA-DRB1∗07:01	132	146	KEHGEPLFSSHMLDL	17.8	3.37
	HLA-DPA1∗01	135	149	GEPLFSSHMLDLSEE	93.6	5.05
	HLA-DPB1∗04:01	135	149	GEPLFSSHMLDLSEE	93.6	5.05
	HLA-DPA1∗01:03	133	147	EHGEPLFSSHMLDLS	46	4.82
	HLA-DPB1∗02:01	133	147	EHGEPLFSSHMLDLS	46	4.82
	HLA-DPA1∗02:01	134	148	HGEPLFSSHMLDLSE	59	6.3
	HLA-DPB1∗01:01	134	148	HGEPLFSSHMLDLSE	59	6.3
	HLA-DPA1∗03:01	135	149	GEPLFSSHMLDLSEE	12	1.14
	HLA-DPB1∗04:02	135	149	GEPLFSSHMLDLSEE	12	1.14
*IRGSIAAAH*	HLA-DRB1∗01:01	81	95	NASIRGSIAAAHYIR	31.5	15.98
	HLA-DRB1∗04:01	81	95	NASIRGSIAAAHYIR	86.5	7.02
	HLA-DRB5∗01:01	81	95	NASIRGSIAAAHYIR	7.3	1.55
	HLA-DQA1∗01:02	80	94	QNASIRGSIAAAHYI	59.3	3.74
	HLA-DQB1∗06:02	80	94	QNASIRGSIAAAHYI	59.3	3.74
	HLA-DQA1∗05:01	81	95	NASIRGSIAAAHYIR	4.6	0.27
	HLA-DQB1∗03:01	81	95	NASIRGSIAAAHYIR	4.6	0.27
*YQAGNVVLS*	HLA-DRB1∗01:01	227	241	HGVYQAGNVVLSPEI	19.7	11.15
	HLA-DRB1∗09:01	227	241	HGVYQAGNVVLSPEI	80.9	5.58
	HLA-DQA1∗01:02	227	241	HGVYQAGNVVLSPEI	91.3	6.42
	HLA-DQB1∗06:02	227	241	HGVYQAGNVVLSPEI	91.3	6.42
	HLA-DQA1∗05:01	224	238	GNVHGVYQAGNVVLS	7.9	0.96
	HLA-DQB1∗03:01	224	238	GNVHGVYQAGNVVLS	7.9	0.96
*VVAALEAAR*	HLA-DRB1∗03:01	41	55	SSTVVAALEAARDAK	50.9	2.91
	HLA-DRB1∗09:01	41	55	SSTVVAALEAARDAK	95.1	6.59
	HLA-DRB5∗01:01	41	55	SSTVVAALEAARDAK	15	3.71
	HLA-DQA1∗01:02	40	54	SSSTVVAALEAARDA	38.1	1.93
	HLA-DQB1∗06:02	40	54	SSSTVVAALEAARDA	38.1	1.93
	HLA-DQA1∗05:01	42	56	STVVAALEAARDAKS	16.2	2.87
	HLA-DQB1∗03:01	42	56	STVVAALEAARDAKS	16.2	2.87
*IAPAYGIPV*	HLA-DRB1∗01:01	94	108	IRSIAPAYGIPVVLH	12.1	6.74
	HLA-DRB1∗07:01	91	105	AHYIRSIAPAYGIPV	34.1	6.37
	HLA-DRB1∗15:01	94	108	IRSIAPAYGIPVVLH	79.2	8.07
	HLA-DQA1∗05:01	94	108	IRSIAPAYGIPVVLH	16.5	2.94
	HLA-DQB1∗03:01	94	108	IRSIAPAYGIPVVLH	16.5	2.94

**Table 6:** Amino acid composition of the protein (fructose bisphosphate aldolase) with their number and molecular weight (Mol%) using BioEdit software version 7.0.5.3.

Amino acid	Number	Mol%	Amino acid	Number	Mol%
Ala A	39	10.80	Leu L	23	6.37
Cys C	3	0.83	Met M	9	2.49
Asp D	21	5.82	Asn N	19	5.26
Glu E	26	7.20	Pro P	15	4.16
Phe F	14	3.88	Gln Q	8	2.22
Gly G	30	8.31	Arg R	10	2.77
His H	12	3.32	Ser S	23	6.37
Ile I	20	5.54	Thr T	17	4.71
Lys K	25	6.93	Val V	29	8.03
Trp W	3	0.83	Tyr Y	15	4.16

**Table 7:** Docking results of the most promiscuous epitopes that show the best binding affinity.

Epitope	Binding MHC molecule	Binding energy (*Δ*G^∗^ kcal/mol)
*AVHEALAPI*	HLA-A∗02:06	-15.8010
*KYFKRMAAM*	HLA-A∗02:06	-20.5935
*QTSNGGAAY*	HLA-A∗02:06	-30.5467
*RMAAMNQWL*	HLA-A∗02:06	-20.6392
*YFKEHGEPL*	HLA-A∗02:06	-16.7505
*IRGSIAAAH*	HLA-DRB1∗01:01	-20.6557
*LFSSHMLDL*	HLA-DRB1∗01:01	-25.5732
*VVAALEAAR*	HLA-DRB1∗01:01	-19.8404

^∗^Global energy: it is the energy required to estimate the strength of association between the epitope within the active.

## Data Availability

The data used to support the findings of this study are available from the corresponding author upon request.
